# Plasma protein fractions in free-living white-tailed eagle (*Haliaeetus albicilla*) nestlings from Norway

**DOI:** 10.1186/s12917-019-2022-6

**Published:** 2019-08-13

**Authors:** Jørgen Flo, Mari Engvig Løseth, Christian Sonne, Veerle L. B. Jaspers, Hege Brun-Hansen

**Affiliations:** 1 Department of Biology, Norwegian University of Science and Technology (NTNU), Høgskoleringen 5, 7491 Trondheim, Norway; 2 Department of Bioscience, Arctic Research Center (ARC), Aarhus University, PO Box 358, Frederiksborgvej 399, DK-4000 Roskilde, Denmark; 3 Department of Basic Sciences and Aquatic Medicine, Norwegian University of Life Sciences (NMBU), 0454 Oslo, Norway

**Keywords:** Capillary electrophoresis, Avian diagnostics, Acute phase protein, Albumin, Globulin

## Abstract

**Background:**

Capillary electrophoresis of plasma proteins has shown great potential as a complementary diagnostic tool for avian species. However, reference intervals for plasma proteins are sparse or lacking for several free-living avian species. The current study reports electrophoretic patterns and concentrations of plasma proteins determined for 70 free-living white-tailed eagle (*Haliaeetus albicilla*) nestlings from two locations in Norway (Steigen and Smøla) in order to establish reference values for this subpopulation using capillary electrophoresis. The nestlings were between 44 and 87 days of age, and the plasma protein concentrations were investigated for age, sex, year (2015 and 2016) and location differences. To our knowledge, this is the first report of reference intervals of plasma proteins analysed by capillary electrophoresis in free-living white-tailed eagle nestlings.

**Results:**

The plasma protein concentrations (% of total protein, mean ± SE) were determined for prealbumin (13.7%, 4.34 ± 0.15 g/L), albumin (46.7%, 14.81 ± 0.24 g/L), α_1_-globulin (2.4%, 0.74 ± 0.03 g/L), α_2_-globulin (11.7%, 3.72 ± 0.06 g/L), β-globulin (15.9%, 5.06 ± 0.08 g/L) and γ-globulin (9.6%, 3.05 ± 0.09 g/L). Significant differences were found between the two locations for prealbumin, α_2_- and γ-globulins. No significant differences were found between the two sampling years or sexes, and no effect of age was found for any of the plasma proteins. However, prealbumin levels were several folds higher than previously reported from adults of closely related birds of prey species. There were no other studies on capillary electrophoresis of nestling plasma available for comparison.

**Conclusion:**

Significant differences were found between sampling locations for prealbumin, α_2_- and γ-globulins, which may indicate differences in inflammatory or infectious status between nestlings at the two locations. Sampling year, sex or age had no significant effect on the plasma protein concentrations. These results provide novel data on plasma protein concentrations by capillary electrophoresis and may be useful for evaluation of health status in free-living white-tailed eagle nestlings.

**Electronic supplementary material:**

The online version of this article (10.1186/s12917-019-2022-6) contains supplementary material, which is available to authorized users.

## Background

Plasma proteins are essential for normal physiological health and provide an important diagnostic tool in human and veterinary medicine [8, 34]. The proteins are important for maintaining osmotic pressure and pH, for transportation of biomolecules, blood clotting, regulation of cellular activity and immune responses [1, 42]. To separate and analyse these proteins, agarose gel- and capillary electrophoresis are two commonly applied methods, which are based on the separation of proteins by an electrical charge. In agarose gel electrophoresis (AGE), the migration matrix for the plasma proteins is a thin layer of agarose gel submerged in buffer. Despite available semi-automated AGE systems, the analysis remains labour-intensive [18]. Capillary electrophoresis (CE) is a fully automated and fast alternative to AGE, using thin silica-fused capillaries as separation matrix instead of agarose gel [18]. When compared to AGE, studies have shown that CE is a reliable method with high reproducibility and repeatability [3, 11, 38]. In mammalian studies, serum is normally used to determine serum proteins while in avian studies, plasma is the preferred sample matrix due to lower sample volumes, easier storage and fluidity [41, 42]. Other methods such as enzyme- and radioimmunoassay, Western blot and mRNA analysis are also available for measuring individual plasma proteins as biomarkers for immune responses. However, these methods often utilise species-specific reagents and may not be available for avian species.

Plasma proteins are most often divided into six fractions; prealbumin, albumin, α_1_-globulins, α_2_-globulins, β-globulins and γ-globulins, depending on their charge and size. Each fraction contains one or more proteins, and especially the β- and γ-fractions contain valuable information for detection and diagnostics of diseases [34]. Inflammatory diseases have been shown to increase levels of the positive acute phase proteins that either migrate in the α-globulin region e.g.: α_2_-macroglobulin and haptoglobin, or in the β-globulin region eg: C reactive protein, serum amyloid A and fibrinogen. Albumin and prealbumin are both negative acute phase proteins and will decrease with inflammation [9, 20]. Antibody responses increase levels of γ-globulins in the blood in exposure to pathogens [20] or as a result of immune mediated disease. A multitude of avian diseases such as aspergillosis [7, 23], sarcocystosis [10], chlamydophilosis, nephritis and hepatitis [8] are known to affect plasma protein levels in birds.

Analyses of plasma proteins may therefore provide important information for health assessment in birds. Studies have shown more variation in electrophoretic patterns among avian, than in mammalian species [4, 41, 44] indicating the need of species-specific reference intervals. Physiological factors such as age and nutrition may also affect protein fractions, although to a lesser degree than inflammation or disease [45]. As top predators, birds of prey can be used as sentinels of environmental pollution [12] or ecosystem health [40], and information on electrophoretic patterns and plasma protein ranges are currently lacking for several birds of prey species.

The white-tailed eagle (*Haliaeetus albicilla*) is a territorial, long-lived bird of prey with an apex position in the food web. Their diet depends on the location of their territories and consists mainly of terrestrial and marine carrion, fish, seabirds and small mammals [15]. Studies have shown that white-tailed eagles are prone to exposure to ecto- and hemoparasites [26, 30]. Post-mortem examinations of white-tailed eagles have shown coccidian and helminth species which reflect parasites commonly found in prey reservoirs [27, 28]. White-tailed eagle nestlings are stationary in their nests during development and thus fed by their parents. As nestlings have an immature immune system during growth, they may be more sensitive to parasite exposure or infections than adults [46].

The aim of the present study was to provide capillary electrophoretic patterns and plasma protein levels for free-living white-tailed eagle nestlings at two locations in Norway (Smøla and Steigen archipelago). Further, we evaluated if protein levels varied depending on location, sex, year, time of day and age at sampling.

## Results

### Description and identification of electrophoretic protein patterns

Six major protein fractions were detected in plasma from white-tailed eagle nestlings. These were prealbumin, albumin, α_1_-globulin, α_2_-globulin, β-globulin and γ-globulin, and their respective concentrations (mean ± standard error (SE), median, range, 95% reference intervals and 90% confidence intervals) found at Smøla and Steigen are provided in Table 1.

**Table 1:** Concentrations and reference intervals^a^ of plasma proteins in Norwegian white-tailed eagle nestlings

SMØLA (*n* = 35)						
Analyte		Mean ± SE	Median	Range	95% RI lower limit (90% CI)	95% RI upper limit (90% CI)
Prealbumin	(g/L)	4.75 ± 0.21	4.36	2.72–7.89	2.62 (2.35–2.98)	7.91 (6.88–9.00)
Albumin	(g/L)	14.66 ± 0.30	14.84	11.52–19.07	11.41 (10.87–12.11)	18.74 (17.59–19.79)
α_1_-globulin	(g/L)	0.71 ± 0.04	0.65	0.36–1.45	0.37 (0.34–0.42)	1.32 (1.09–1.56)
α_2_-globulin	(g/L)	3.56 ± 0.07	3.55	2.77–4.41	2.77 (2.60–2.94)	4.47 (4.21–4.70)
β-globulin	(g/L)	4.98 ± 0.12	4.81	4.03–7.69	3.99 (3.85–4.16)	6.36 (5.59–6.89)
γ-globulin	(g/L)	2.74 ± 0.10	2.64	2.00–4.45	1.88 (1.79–2.00)	4.08 (3.64–4.59)
Total protein	(g/L)	31.40 ± 0.45	31.40	26.00–35.90	25.47 (24.36–26.99)	36.62 (35.44–37.57)
A:G^b^ ratio		1.63 ± 0.03	1.59	1.28–2.09	1.30 (1.25–1.36)	2.09 (1.94–2.25)
A:G^c^ ratio		0.88 ± 0.02	0.87	0.54–1.20	0.61 (0.55–0.69)	1.14 (1.06–1.20)
STEIGEN (*n* = 35)						
Analyte		Mean ± SE	Median	Range	95% RI lower limit (90% CI)	95% RI upper limit (90% CI)
Prealbumin	(g/L)	3.93 ± 0.18	3.78	1.85–6.63	2.10 (1.82–2.44)	6.56 (5.74–7.53)
Albumin	(g/L)	14.97 ± 0.38	14.82	9.71–19.35	10.29 (9.32–11.32)	19.58 (18.25–20.82)
α_1_-globulin	(g/L)	0.79 ± 0.04	0.73	0.42–1.38	0.43 (0.39–0.49)	1.21 (1.08–1.39)
α_2_-globulin	(g/L)	3.88 ± 0.10	3.74	2.80–5.25	2.82 (2.65–3.02)	5.30 (4.82–5.72)
β-globulin	(g/L)	5.13 ± 0.12	5.15	3.51–6.35	3.70 (3.39–4.02)	6.57 (6.20–6.88)
γ-globulin	(g/L)	3.37 ± 0.13	3.30	2.01–5.23	1.96 (1.71–2.27)	5.12 (4.68–5.65)
Total protein	(g/L)	32.06 ± 0.68	32.40	20.30–39.60	24.15 (22.36–26.30)	41.20 (38.40–43.44)
A:G^b^ ratio		1.44 ± 0.02	1.42	1.16–1.76	1.17 (1.12–1.22)	1.78 (1.68–1.86)
A:G^c^ ratio		0.88 ± 0.02	0.87	0.73–1.11	0.70 (0.67–0.73)	1.13 (1.06–1.21)

^a^95% Reference intervals (95% RI) were calculated using a robust method based on Box-Cox transformed data. Confidence intervals (90% CI) around the upper and lower reference limit were obtained through a nonparametric bootstrap

^b^Albumin to globulin ratio: (albumin + prealbumin)/(α_1_ + α_2_+ β + γ globulins)

^c^Albumin to globulin ratio: (albumin)/(prealbumin + α_1_ − + α_2_ − + β − + γ-globulins, [38])

Plasma samples were collected from nestlings at Smøla and Steigen in 2015 and 2016 and samples were combined for each location due to statistically significant differences between locations and not between years (Table 2). The concentrations are in g/L. Additional information regarding reference and confidence intervals for Smøla and Steigen nestlings is found in the supplementary information (Additional file 1: Figure S3 and S4). The percent of total protein content (%) and concentrations of plasma proteins in nestlings sampled in 2015 and 2016 for each location separately can be found in the supplementary information (Additional file 1: Table S2).

**Fig. 1:**
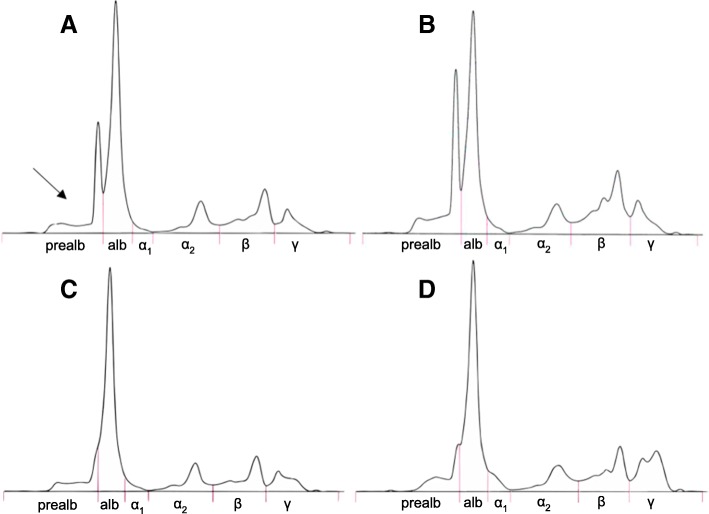
Electrophoretic patterns of plasma protein fractions from white-tailed eagle nestlings from Steigen and Smøla. Protein fractions (from left to right) are prealbumin, albumin, α_1_-globulin, α_2_-globulin, β-globulin and γ-globulin. **a** Typical pattern from Smøla nestlings, with a separate prealbumin peak indicated with a black arrow, **b** Pattern from the nestling with the highest levels of prealbumin and β-globulin. **c** Typical pattern of Steigen nestling, with a “smooth” transition between prealbumin and albumin. **d** Pattern of nestling with the highest levels of γ-globulin

The electrophoretic patterns (Fig. 1) from 80% of the nestlings from Smøla had clear prealbumin peaks shouldering off from the albumin fraction (Fig. 1a), compared to 26% of the nestlings from Steigen. Most of the nestlings from Steigen had no prealbumin shoulder (Fig. 1c), or a small shoulder (Fig. 1d). The electrophoretic patterns for the nestlings with the highest levels of β-globulin and γ-globulin are shown in Fig. 1b and d, respectively.

### Influence of sampling locations, years, sex, time of day and age on plasma protein concentrations

When the two years were combined, statistically significant differences were detected between locations for prealbumin, α_2_- and γ-globulins (*p* < 0.01), with higher levels of prealbumin in nestlings from Smøla and higher levels of α_2_- and γ-globulins in nestlings from Steigen (Table 2). No statistically significant differences were found between females and males, and the age of the nestlings and the time of day at sampling did not affect the variation of the plasma proteins (Table 2).

**Table 2:** Statistical differences between plasma protein concentrations between locations, years, sex, nestling age and sampling time

		Steigen - Smøla		2015–2016		Age		Sex		Time of day	
		*n*: 35–35		*n*: 27–43		*n*: 70		*n*: 35–35		*n*: 70	
		F-value	*p*-value	F-value	*p*-value	F-value	*p*-value	F-value	*p-*value	F-value	*p-*value
Prealbumin	(g/L)	7.41	0.01*	2.19	0.15	0.24	0.63	1.29	0.27	0.08	0.77
Albumin	(g/L)	0.10	0.75	3.55	0.07	0.80	0.38	0.84	0.37	0.09	0.76
α_1_-globulin	(g/L)	2.89	0.10	0.22	0.64	0.75	0.40	3.64	0.07	0.90	0.35
α_2_-globulin	(g/L)	4.60	0.04*	3.31	0.08	0.08	0.79	0.22	0.65	0.01	0.91
β-globulin	(g/L)	0.70	0.41	0.27	0.61	0.01	0.93	0.32	0.58	0.40	0.54
γ-globulin	(g/L)	11.79	< 0.01*	0.03	0.86	0.03	0.87	1.30	0.27	3.02	0.10
Total protein	(g/L)	0.48	0.49	1.39	0.25	0.49	0.49	1.26	0.28	0.02	0.88
A:G^a^ ratio		17.37	< 0.01*	0.09	0.77	2.18	0.15	0.22	0.65	0.39	0.85
A:G^b^ ratio		0.14	0.71	3.75	0.06	0.70	0.41	0.80	0.38	0.65	0.43

^a^Albumin to globulin ratio: (albumin + prealbumin)/(α_1_ + α_2_ + β + γ globulins)

^b^Albumin to globumin ratio: (albumin)/(prealbumin + α_1_- + α_2_- + β- + γ-globulins, [38])

Statistically significant differences are marked with asterisks (*)

No statistically significant differences were found between the two years when locations were combined (Table 2), however, inter-annual differences were found for albumin in Steigen with higher concentrations in 2016 than 2015 (F_(17,1)_ = 5.87, *p* = 0.02). Statistically significant intra-annual differences were also found between the two locations, with higher concentrations of prealbumin at Smøla and α_1_-globulins at Steigen in 2015 (F_(17,1)_ = 6.13–6.21, *p* = 0.02). In 2016, significantly higher concentrations of α_2_- and γ-globulins were found in Steigen compared to Smøla. When the two sexes were separated, statistically significant differences between the locations for prealbumin, α_2_- and γ-globulins were found for females (F_(27,1)_ = 4.46–13.84, *p* = 0.04), but not males (*p* > 0.05). Statistically significant differences between years were also found for prealbumin and albumin for females only (F_(27,1)_ = 13.33 and 5.14, *p* < 0.03, respectively).

## Discussion

The electrophoretic patterns found for the white-tailed eagle nestlings in the present study resembled those found for black kite (*Milvus migrans*) by CE analyses [38] and bald eagle (*Haliaeetus leucocephalus*) nestlings by AGE analyses [41]. The concentrations of total protein, albumin, α_2_-, β- and γ-globulins were also similar to those observed by AGE of plasma from healthy stellar’s sea eagle (*Haliaeetus pelagicus*) and bald eagle adults [41], and within the range of concentrations observed in free-living nestling peregrine falcons (*Falco peregrinus*) by cellulose acetate electrophoresis [29]. Compared to these previous studies, the most notable differences were the lower levels of α_1_-globulin and higher levels of prealbumin in the present study.

The α_1_-globulin fraction is often not detectable in plasma of healthy birds from most avian species, while plasma from raptorial species show a high-amplitude fraction branching off the albumin spike [44]. The mean concentration of α_1_-globulin in the present study was 2.4% of the total protein content (Additional file 1: Table S2), which is lower than what has been found for α_1_-globulins in peregrine falcon nestlings (6.4% of the total protein content, [29]). The concentrations of the present study were close to 1.4 g/L or 4% of total protein as found in plasma of adult bald eagles using AGE [24]. These differences are most likely due to species differences although the age of the birds may also interfere with the plasma protein concentrations.

The concentrations of prealbumin in the present white-tailed eagle nestlings constituted 13.7% of the total plasma protein content (Additional file 1: Table S2), which is similar to concentrations found for healthy juvenile gyr–saker hybrid falcons (*Falco rusticolus* x *Falco cherrug*) [14], adult Spanish imperial eagles (*Aquila adalberti*) and golden eagles (*Aquila chrysaetos*) [35]. These prealbumin concentrations are higher than those reported in studies on bald eagles showing values of 2.1–4.0% [24, 41]. A study by Roman et al. [38] found significant correlations for albumin, β- and γ-globulin when comparing protein fractions from agarose gel electrophoresis and capillary electrophoresis in plasma from several bird species. Interestingly, they also found significant correlations between prealbumin values obtained by capillary electrophoresis and α-globulin values from agarose gel electrophoresis, which can imply that some proteins from the α-region may migrate to the prealbumin region when using capillary electrophoresis. This may possibly explain the high levels of prealbumin and low levels of α-globulin in the present study compared to Jones et al. [24] and Tatum et al. [41] which used agarose gel electrophoresis. Cray et al. [6] demonstrated differences in protein fractions of avian plasma due to different electrophoretic systems. Hence, the analytical methods used to determine concentrations of plasma proteins should be considered when comparing results among studies. However, the study of Fischer et al. [14] was also performed using AGE, which indicates that there may also be species-specific differences regarding prealbumin in birds of prey.

### Plasma proteins and potential disease

Concentrations of plasma proteins can show a large variation in birds exposed to disease, inflammation, variable temperatures and different diets. The α-, β- and γ-globulins are known to include positive acute phase proteins or immunoglobulins and their concentrations often increase in response to an infection or inflammation, while prealbumin and albumin are negative acute phase proteins as their concentrations often decrease [8, 9]. The two sample locations, Steigen and Smøla, are breeding areas for migrating birds such as geese, which may transport diseases and ectoparasites to these locations [2, 25]. The samples from the present study were taken from free-living nestlings, which may have been exposed to a variety of diseases and parasites through prey or their parents while in the nests. The significantly higher concentrations of α_2_- and γ-globulin, and lower concentrations of prealbumin in Steigen compared to Smøla, may indicate that some of the nestlings from Steigen could have had an acute immune response to an infection or inflammation. Ectoparasites were present in the plumage of some nestlings, however, there were no differences regarding the locations. One of the field workers were infected with the endoparasite Cryptosporidium sp. after fieldwork in 2016, which most likely originated from the nestlings. However, it is not possible to know the location of origin. All the nestlings were also screened for avian influenza and the test was negative at both locations (Lee et al., in submission). Still, variation in exposure to infections or inflammations may explain the observed temporal and spatial differences in plasma proteins.

A study on prefledging caspian terns (*Sterna caspia*) and herring gulls (*Larus argentatus*) also found significant differences in positive APPs (α- and β-globulins) between sampling locations [21]. Although their study design and analytical method differed from the present study, their results support the notion that geographical location or underlying factors at these locations may affect plasma protein concentrations. Løseth et al. [31] have shown indications of a significantly higher input of terrestrial species in the diet of nestlings from Smøla than Steigen which may offer another explanation to the spatial variation the plasma proteins.

### Age of the nestlings

Age-related differences have been reported for plasma protein profiles in avian species [44]. A previous study investigated the relationship between protein fractions and age in captive bald eagles (aged 6–43 years) and found a significant increase of total protein and β-globulin with age [24]. Such differences were not found in the current study (Table 2), most likely due to the short age span of nestlings, which were only 6–12 weeks old. However, the prealbumin in the plasma of the sampled nestlings were several folds higher relative to values reported for adult birds from other raptor-species [24, 41]. Prealbumin is a carrier protein for growth hormones [33] and thyroid hormones [36], and since nestlings are in a stage of rapid growth it might offer an explanation for the relatively high levels of prealbumin observed in the current study. This proposed explanation is further supported by a study on wild peregrine falcon nestlings using cellulose acetate electrophoresis [29], where the authors found prealbumin to constitute 8.35% of the total plasma protein, compared to 13.7% in the present white-tailed eagle nestlings (Additional file 1: Table S2).

### Time of day

Circadian rhythms have been reported to affect concentrations of plasma proteins, various hormones and blood clinical-chemical parameters in birds [13, 17, 39]. The photoperiod or light cycle during the day is an important cue for circadian rhythms, as such studies advice to collect blood samples mid-day when the light is relatively stable [17, 39]. The present study found no effect of sampling time of day on the variation on the plasma proteins (Table 2, Additional file 1: Figure S6), most likely due to the long light period (midnight sun) at both locations during June–July.

To summarize, capillary electrophoresis may offer a rapid and efficient health assessment of avian species, given that reference intervals for wild birds become available. As the plasma protein levels from the present study varied significantly compared to previous studies of related bird of prey species, it is likely that the presented levels are species-specific. Thus, more baseline data on avian species are needed in order to use capillary electrophoresis as a diagnostic tool for avian wildlife.

## Conclusion

The present study provides the first report of protein fractions in plasma from free-living white-tailed nestling eagles. The variation in plasma protein levels was primarily a result of sample location, and variations related to sex, age, time of day and year of sampling were less indicative. The values provided for these Norwegian free-living nestlings can be useful as reference intervals for further evaluation of health status of white-tailed eagle nestlings and may potentially be used as biomarkers of health.

## Methods

### Field sampling

White-tailed eagle nestlings were sampled in 2015 (*n* = 27) and 2016 (*n* = 43) at Smøla (63.35°N; 8.03°E) and Steigen (67.93°N; 14.98°E) in Norway as described in Løseth et al. (2019a,b, Additional file 1: Figure S1). Sex was determined by comparing tarsus depth and bill height with wing length, as females are significantly larger than males [22]. The age of the nestlings ranged from 44 to 87 days and was calculated based on tail feather length as described in [31]. The body mass of the nestlings ranged from 3.7 to 6.7 kg. Detailed information regarding age and body mass can be found in the supplementary information (Additional file 1: Table S1). A limited physical examination was done to confirm gross clinical health. There were no visible signs of wounds, swelling or of diarrhoea by faeces stuck to the cloaca. The nestlings seem to be in good condition based on their body condition index ranging from − 1.30 to 1.42 [31] and other research (e.g. blood clinical chemistry; [47]). All nestlings were sampled from each nest (1–3 nestlings) and blood was drawn by brachial venepuncture using a heparinized vacutainer (Venosafe® BD, 9 mL) and a 23-gauge needle (0.6 mm). The majority of blood samples were collected during daylight between 10:00 and 18:00, while three samples were collected between 20:00 and 22:00 (midnight sun). Samples were stored in cooling bags until centrifugation, and the plasma was transferred and stored in sterile cryotubes at − 20 °C until analysis (up to 8 months after sampling). The sampling was in accordance with the regulations of the Norwegian Animal Welfare Act and approved by the Norwegian Food Safety Authority (Mattilsynet; 2015/6432 and 2016/8709). Previous studies of these white-tailed eagle nestlings have found low plasma concentrations of organohalogenated contaminants, which mainly reflect background contamination in their local environments [31, 32]. The plasma protein levels from these nestlings may therefore present suitable to reference ranges for other free-living nestlings.

### Biuret test

Total protein was determined using Siemens ADVIA 1800 automated chemistry analyzer according to the product manual (Siemens Healthineers, Germany). In brief, a volume of 30 μL plasma was diluted with 120 μL saline solution (physiological 0.9%) and 17.5 μL of the diluted sample was transferred to a test tube for further extraction with two reagents. Reagent 1 (68.5 μL, TP R1, Lot: 129TP) contained sodium hydroxide (400 mmol/L) and Na-K-tartrate (92 mmol/L), while reagent 2 (68.5 μL, TP R2, Lot: 130TP) contained the two prior ingredients as well as potassium iodide (60 mmol/L) and cupric sulphate (24 mmol/L) (Advia Chemistry, Siemens Healthineers, Germany). The cupric ions in reagent 2 interact with the protein peptide bonds and form a purple complex. The intensity of the purple colour is then used to measure the amount of total protein in the sample by spectrophotometry at 545 nm [43].

### Capillary protein electrophoresis

Plasma protein fractions were obtained with Capillarys 2 instrument (Sebia®, Lisses, France), using CAPILLARYS PROTEIN(E) 6 kit (PN 2003) according to the manufacturer’s instructions. Plasma (40 μL) was added to 8 well dilution segments (8 sample tubes and up to 13 racks) and diluted 1:5 with migration buffer (160 μL, alkaline buffer pH 9.9, Capillarys Protein(e) 6, Lot: 18055/01, Sebia®). Samples were then injected by aspiration in the anodic end of silica glass capillaries with constant temperature (Peltier effect) and voltage for separation. The protein patterns were detected by absorption photometry (200 nm, deuterium lamp, Sebia®) at the cathodic end of the capillaries and patterns were visualized with PHORESIS software (version 8.6.3). The capillaries were then immediately washed with a wash solution containing sodium hydroxide and distilled water (Capillarys, Lot: 22066/01, Sebia®) and prepared for the next analysis. Each fraction was quantified by the area under the electrophoretic curve as a percentage of total protein, and identification of each band was done manually.

### Quality assurance and control

Before determining total protein, ADVIA 1800 was calibrated (Siemens chemistry calibrator, REF 09784096) using canine serum as a laboratory control (Siemens healthineers, Germany). Two liquid human serum-based controls (BioRad 1, BioRad 3, Bio-Rad®) were also assessed weekly (with lab assurance expressed as coefficient of variation: CV < 2%). For capillary electrophoresis, a set of eight controls (Human control serum, REF 4785, Sebia®) was assessed before running the white-tailed eagle samples. For all individuals, the protein fractions were interpreted by visual inspection of the graphs (Fig. 1). To assure the optimal division of protein fractions the electrophoretic patterns from AGE of adult bald eagles were used as a reference [41]. The divisions between prealbumin, albumin and α_1_-globulin were assessed multiple times to assure consistency between samples as these were the most challenging fractions to divide.

### Statistics

Analyses were primarily performed in the statistical program R v 3.2.5 [37]. Normality of the protein fraction was assessed by Shapiro-Wilk’s test and by visual inspection of quantile-quantile plots. Non-normal distributions were found for prealbumin, α_1_-, β- and γ-globulin, and these variables were log_e_-transformed to ensure normality before parametric tests were applied. Albumin:Globulin ratios (A:G ratio) were calculated both by the traditional way by dividing the sum of albumin and prealbumin by the sum of globulins (α_1_, α_2_, β and γ) [7, 8], and as proposed by Roman et al. [38] by dividing albumin by the sum of prealbumin and the globulins (Table 1). Reference intervals (RI) was obtained according to guidelines and recommendations from the 2008 Clinical Laboratory and Standards Institute [5] and the American Society for Veterinary Clinical Pathology [16]. Individual reference intervals for Smøla and Steigen, on combined years as there were no statistically significant differences between years (Table 2), are given in Table 1. The calculation of RI was performed using Reference Value Advisor v.2.1 [19] and Microsoft® Excel® for Office 365 v.1905. In brief, the distributions were assessed by quantile-quantile plots and Anderson-Darling Test, and outliers were assessed with histograms and Tukey test. The data was Box-Cox transformed and RI upper and lower limits were obtained through a non-parametric bootstrap. Retention of data was prioritized due to a low sample size (*n* = 35 for each location) and histograms are provided in the supplementary information where median value, 95% RI and 90% CI are shown for each plasma protein histogram (Additional file 1: Figure S3 and S4). Linear mixed effect models and analyses of variance (lme-ANOVA) were used to investigate differences in plasma protein concentrations between years, locations, and sex, and to investigate if the time of day and the nestling’s age at sampling could influence the variation. Nest was included as a random factor to control for the variation between nestlings within the same nest for all statistical analyses [31]. Statistical significance level was set to α = 0.05 for all tests. The percent of total protein content for each plasma protein can be found in Additional file 1: Figure S5 and Additional file 1: Table S2 in the supplementary information.

## Additional file

Additional file 1: **Table S1.** Body mass and age of white-tailed eagle nestlings. **Table S2.** Plasma protein concentrations in white-tailed eagle nestlings. **Figure S1.** Map of Norway displaying the two sampling locations. **Figure S2.** Scatterplot between age and plasma protein levels. **Figure S3.** Histograms of plasma protein levels and 95% RI (90% CI) in nestlings from Smøla. **Figure S4.** Histograms of plasma protein levels and 95% RI (90% CI) in nestlings from Steigen. **Figure S5.** Percentages of total protein content for each plasma protein fraction. **Figure S6.** Scatterplot between time of day at sampling and plasma protein levels. (DOCX 815 kb)

## Data Availability

The datasets used and/or analysed during the current study are available from the corresponding author on reasonable request.

## Abbreviations

A:G ratio: Albumin to globulin ratio
AGE: Agarose gel-electrophoresis
CE: Capillary electrophoresis
