# Supplementary material for: Plasma protein fractions in free-living white-tailed eagle (Haliaeetus albicilla) nestlings from Norway
Source: BMC Vet Res. 2019 Aug 13;15:290. doi: 10.1186/s12917-019-2022-6 (PMC6693235; doi:10.1186/s12917-019-2022-6)
Supplement: Supplementary file 1 — Table S1. Body mass and age of white-tailed eagle nestlings. Table S2. Plasma protein concentrations in white-tailed eagle nestlings. Figure S1. Map of Norway displaying the two sampling locations. Figure S2. Scatterplot between age and plasma protein levels. Figure S3. Histograms of plasma protein levels and 95% RI (90% CI) in nestlings from Smøla. Figure S4. Histograms of plasma protein levels and 95% RI (90% CI) in nestlings from Steigen. Figure S5. Percentages of total protein content for each plasma protein fraction. Figure S6. Scatterplot between time of day at sampling and plasma protein levels. (DOCX 815 kb) [file 12917_2019_2022_MOESM1_ESM.docx]

Plasma protein fractions in free-living white-tailed eagle (*Haliaeetus albicilla*) nestlings in Norway

Jørgen Flo^a^, Mari Engvig Løseth^a^, Christian Sonne^b^, Veerle L. B. Jaspers^a^ and Hege Brun-Hansen^c^

Affiliation:

^a^ Department of Biology, Norwegian University of Science and Technology (NTNU), Høgskoleringen 5, 7491 Trondheim, Norway

^b^ Department of Bioscience, Faculty of Science and Technology, Arctic Research Center (ARC), Aarhus University, Frederiksborgvej 399, PO Box 358, DK-4000 Roskilde, Denmark

^c^ Department of Basic Sciences and Aquatic Medicine, Norwegian University of Life Sciences (NMBU), 0454 Oslo, Norway

Number of pages: 5

Number of tables: 2

Number of figures: 6

Contents:

Table S1: Body mass and age of white-tailed eagle nestlings

Table S2: Plasma protein concentrations in white-tailed eagle nestlings

Figure S1: Map of Norway displaying the two sampling locations

Figure S2: Scatterplot between age and plasma protein levels

Figure S3: Histograms of plasma protein levels and 95% RI (90% CI) for nestlings from

Smøla

Figure S4: Histograms of plasma protein levels and 95% RI (90% CI) for nestlings from

Steigen

Figure S5: Percentages of total protein content for each plasma protein fraction

Figure S6: Scatterplot between time of day at sampling and plasma protein levels

**Table S1: Body mass and age of white-tailed eagle nestlings from Smøla and Steigen, 2015 and 2016.**

| SMØLA | 2015 | | | | | | | | 2016 | | | | | | | |
| --- | --- | --- | --- | --- | --- | --- | --- | --- | --- | --- | --- | --- | --- | --- | --- | --- |
|  | n | AM | ± | SE | Median | Range | | | n | AM | ± | SE | Median | Range | | |
| Female mass (kg) | 7 | 6107 | ± | 213 | 6000 | 4994 | - | 6650 | 10 | 5660 | ± | 129 | 5600 | 5000 | - | 6200 |
| Male mass (kg) | 6 | 4825 | ± | 159 | 4975 | 4200 | - | 5200 | 12 | 4496 | ± | 119 | 4550 | 3650 | - | 5300 |
| Age (days) | 13 | 79 | ± | 2 | 81 | 65 | - | 87 | 22 | 66 | ± | 2 | 67 | 52 | - | 82 |
| STEIGEN | 2015 | | | | | | | | 2016 | | | | | | | |
|  | n | AM | ± | SE | Median | Range | | | n | AM | ± | SE | Median | Range | | |
| Female mass (kg) | 8 | 4994 | ± | 180 | 5025 | 4150 | - | 5950 | 10 | 5505 | ± | 97 | 5475 | 5000 | - | 6000 |
| Male mass (kg) | 6 | 4330 | ± | 227 | 4350 | 3650 | - | 4950 | 11 | 4468 | ± | 86 | 4500 | 3850 | - | 5000 |
| Age (days) | 14 | 63 | ± | 5 | 65 | 44 | - | 85 | 21 | 68 | ± | 2 | 71 | 50 | - | 82 |

**Table S2:** **Plasma protein concentrations in Norwegian white-tailed eagle nestlings from Steigen and Smøla, 2015 and 2016.**

|  |  | SMØLA |  |  |  | STEIGEN |  |  |
| --- | --- | --- | --- | --- | --- | --- | --- | --- |
|  | 2015 |  | 2016 |  | 2015 |  | 2016 |  |
|  | *n* = 13 |  | *n* = 22 |  | *n* = 14 |  | *n* = 21 |  |
| Analyte | Mean ± SE | Min – Max | Mean ± SE | Min – Max | Mean ± SE | Min – Max | Mean ± SE | Min – Max |
| Prealbumin | 5.24 ± 0.41 | 2.72 – 7.89 | 4.46 ± 0.22 | 2.83 – 6.62 | 4.05 ± 0.15 | 2.89 – 4.88 | 3.84 ± 0.29 | 1.85 – 6.63 |
|  | 16.4% |  | 14.3% |  | 13.3% |  | 11.6% |  |
| Albumin | 14.55 ± 0.49 | 11.87 – 17.23 | 14.72 ± 0.38 | 11.52 – 19.07 | 13.70 ± 0.35 | 10.64 – 15.18 | 15.82 ± 0.52 | 9.71 – 19.35 |
|  | 45.7% |  | 47.3% |  | 45.2% |  | 47.6% |  |
| α_1_-globulin | 0.65 ± 0.05 | 0.43 – 1.01 | 0.74 ± 0.05 | 0.36 – 1.45 | 0.86 ± 0.07 | 0.45 – 1.38 | 0.73 ± 0.05 | 0.42 – 1.34 |
|  | 2.0% |  | 2.4% |  | 2.8% |  | 2.2% |  |
| α_2_-globulin | 3.47 ± 0.11 | 2.77 – 4.03 | 3.61 ± 0.09 | 2.88 – 4.41 | 3.59 ± 0.10 | 2.96 – 4.21 | 4.08 ± 0.14 | 2.80 – 5.25 |
|  | 10.9% |  | 11.6% |  | 11.8% |  | 12.3% |  |
| β-globulin | 5.16 ± 0.25 | 4.12 – 7.68 | 4.88 ± 0.12 | 4.03 – 6.06 | 4.83 ± 0.12 | 4.07 – 5.68 | 5.34 ± 0.16 | 3.51 – 6.35 |
|  | 16.2% |  | 15.7% |  | 15.9% |  | 16.1% |  |
| γ-globulin | 2.79 ± 0.12 | 2.22 – 3.50 | 2.71 ± 0.14 | 2.00 – 4.45 | 3.31 ± 0.21 | 2.21 – 5.23 | 3.40 ± 0.16 | 2.01 – 4.52 |
|  | 8.8% |  | 8.7% |  | 10.9% |  | 10.2% |  |
| Total protein | 31.87 ± 0.54 | 28.10 – 34.80 | 31.12 ± 0.67 | 26.00 – 35.90 | 30.34 ± 0.65 | 25.10 – 33.80 | 33.21 ± 0.98 | 20.30 – 39.60 |
| A:G^a^ ratio | 1.65 ± 0.05 | 1.39 – 2.09 | 1.62 ± 0.04 | 1.28 – 2.05 | 1.42 ± 0.03 | 1.16 – 1.68 | 1.46 ± 0.04 | 1.20 – 1.76 |
| A:G^b^ ratio | 0.86 ± 0.05 | 0.54 – 1.20 | 0.90 ± 0.02 | 0.76 – 1.13 | 0.83 ± 0.02 | 0.73 – 0.93 | 0.92 ± 0.02 | 0.76 – 1.12 |

^a^Albumin to globulin ratio: (albumin + prealbumin)/(α_1_ + α_2_+ β + γ globulins)

^b^Albumin to globulin ratio: (albumin)/(prealbumin + α_1_-+ α_2_-+ β-+ γ-globulins, Roman et al., 2013)

Mean (± SE) and range of plasma protein concentrations (g/L) and A:G ratios for white-tailed eagle nestlings sampled at Smøla and Steigen in 2015 and 2016, separately. The percentage of the total protein content is also provided for each plasma protein fraction (%).


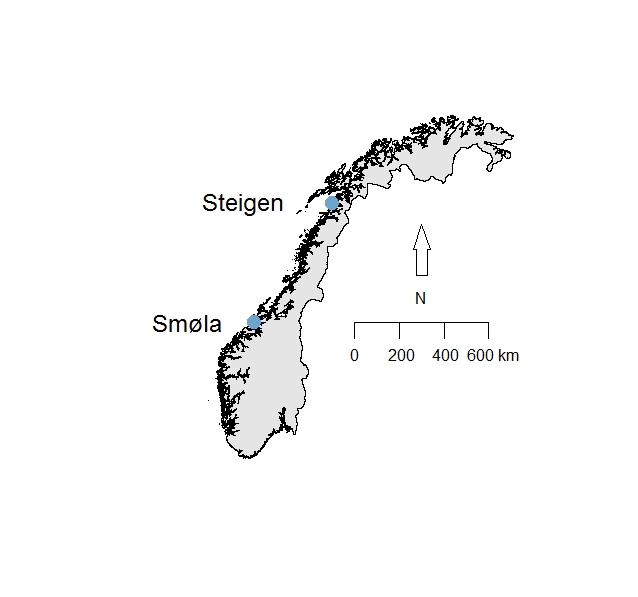


**Figure S1:** Map of Norway displaying the two sampling locations in the present study, Smøla and Steigen.


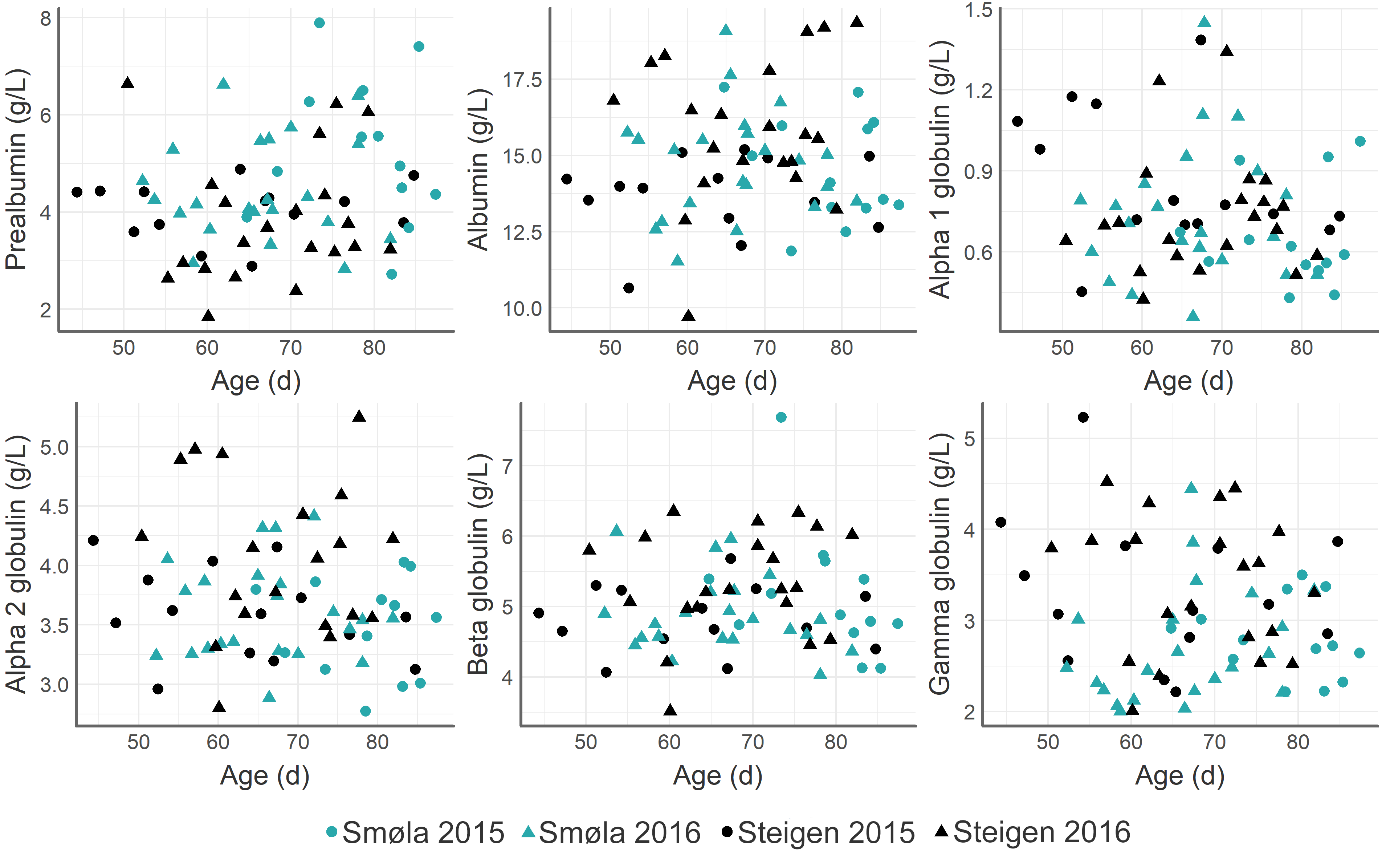


**Figure S2:** Scatterplot between age (days) and protein fractions (g/L) in plasma from white-tailed eagles (n = 70) sampled at Smøla and Steigen Norway, in 2015 and 2016.

**SMØLA**


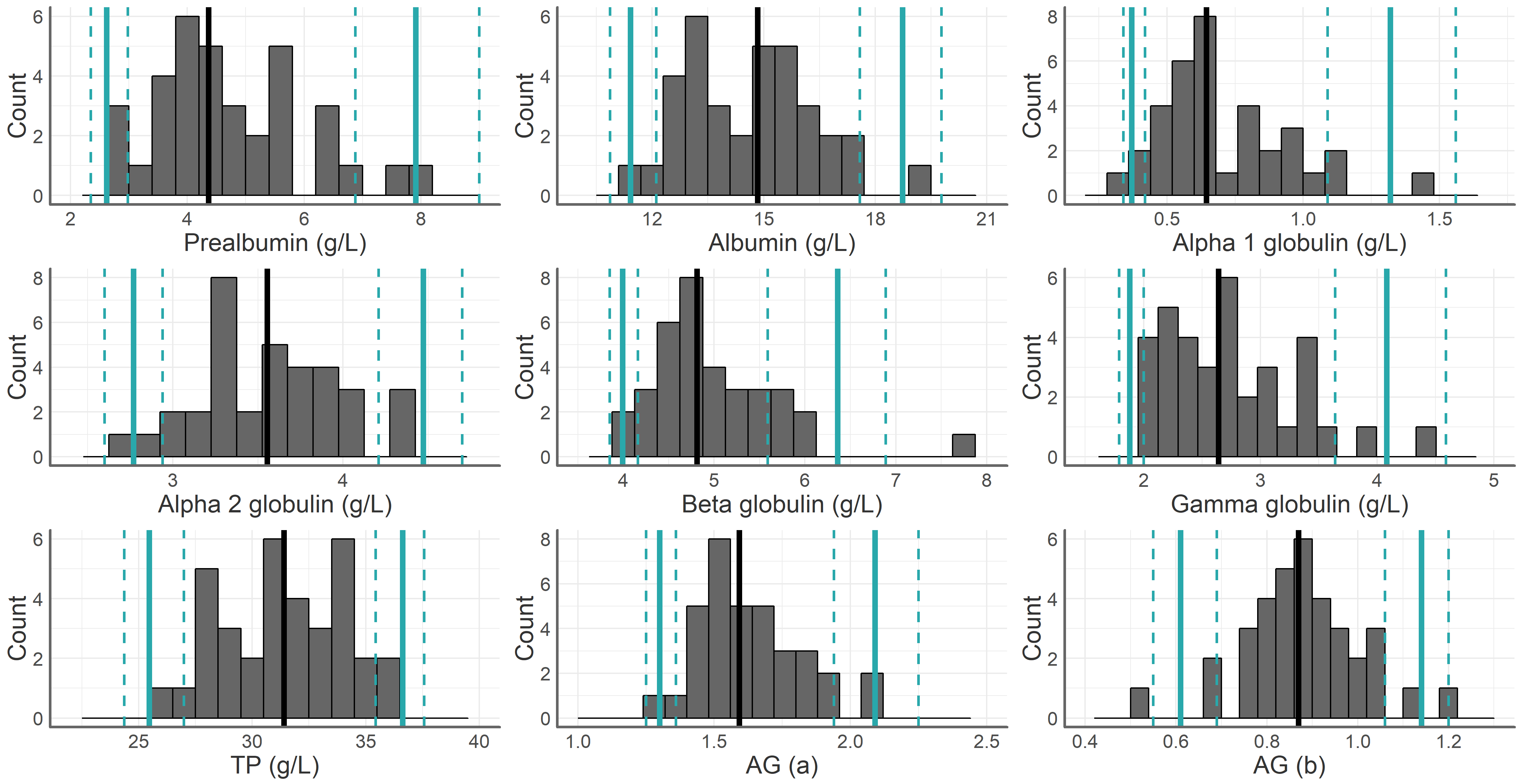


**Figure S3:** Histogram of protein fractions (g/L), total protein (g/L) and A:G ratio, 95% RI limits (solid blue lines), 90% CI around RI limits (dashed blue lines) and median (black lines) in plasma from white-tailed eagle nestlings (n=35) sampled at Smøla, 2015 and 2016.

**STEIGEN**


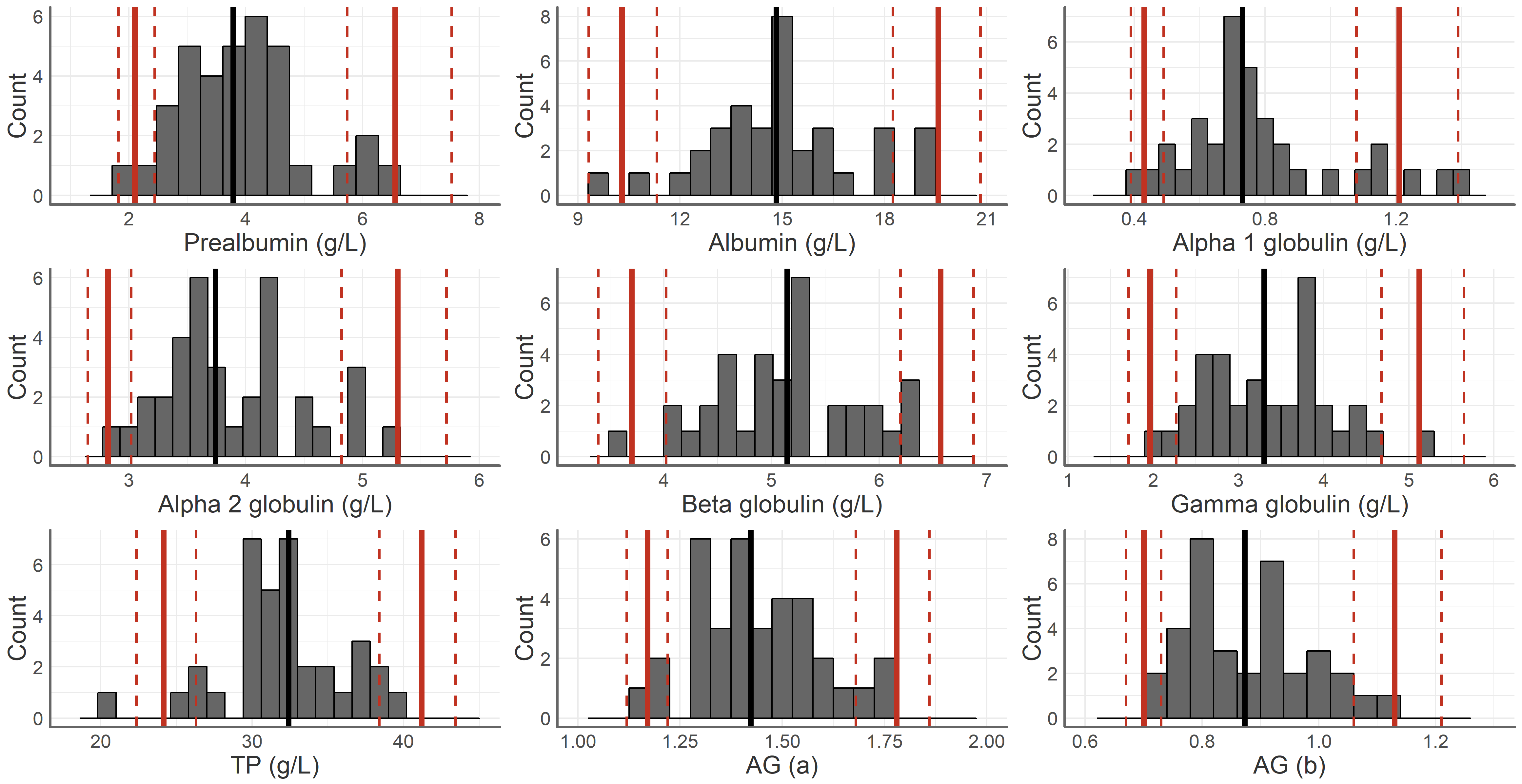


**Figure S4:** Histogram of protein fractions (g/L), total protein (g/L) and A:G ratio, 95% RI limits (solid blue lines), 90% CI around RI limits (dashed blue lines) and median (black lines) in plasma from white-tailed eagle nestlings (n=35) sampled at Steigen, 2015 and 2016.

**Figure S5:** Percentages of total protein for each plasma protein content in plasma of white-tailed eagle nestlings (n = 70) from Smøla and Steigen, 2015 and 2016.


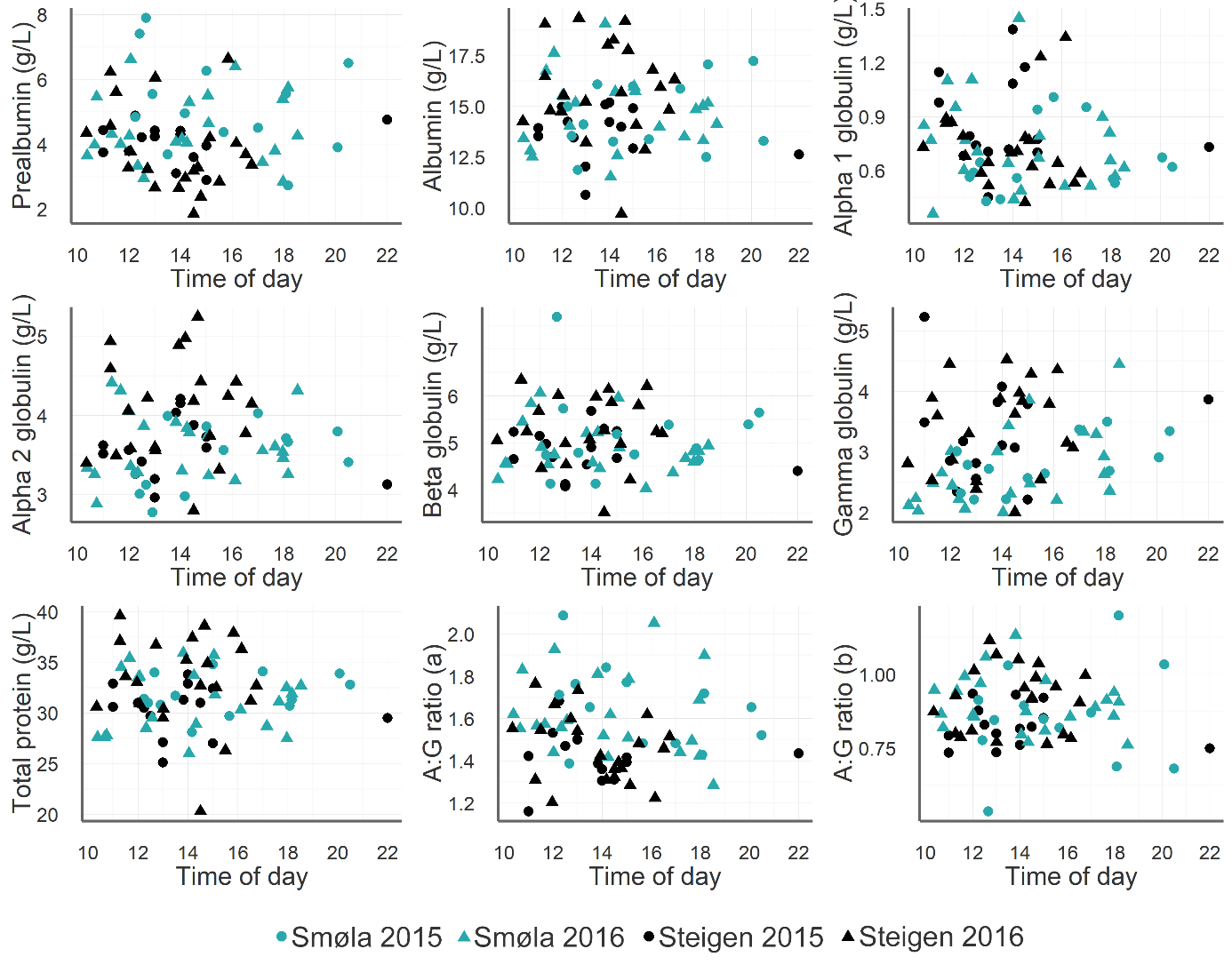


**Figure S6:** Plasma protein levels of white-tailed eagle nestlings in relation to sampling time of day at Smøla and Steigen in 2015 and 2016.
